# Depression and Anxiety After Radiation-Induced Brain Injury: A Review of Current Research Progress

**DOI:** 10.3390/curroncol32080419

**Published:** 2025-07-26

**Authors:** Feng Yang, Rundong Liu, Xiaohong Peng, Na Luo, Min Fu, Wenjun Zhu, Qianxia Li, Guangyuan Hu

**Affiliations:** Department of Oncology, Tongji Hospital, Tongji Medical College, Huazhong University of Science and Technology, Wuhan 430030, China; d202382345@hust.edu.cn (F.Y.);

**Keywords:** tumor treatment complications, radiation-induced brain injury, affective disorder, anxiety, depression

## Abstract

Radiation therapy serves as a fundamental treatment for primary and metastatic brain tumors, whether used alone or combined with surgery and chemotherapy. Despite its oncological efficacy, this treatment paradigm frequently induces radiation-induced brain injury (RBI), a progressive neuropathological condition characterized by structural and functional damage to healthy cerebral parenchyma. Patients with RBI frequently develop affective disorders, particularly major depressive disorder and generalized anxiety disorder, which profoundly impair psychosocial functioning and quality of life. The pathophysiology involves complex mechanisms such as neuroinflammation, oxidative stress, blood–brain barrier disruption, and white matter damage. Current management strategies include antidepressants, corticosteroids, and neuroprotective agents, while emerging therapies targeting neuroinflammation and neural repair show promise. This review comprehensively examines the pathogenesis of RBI-related affective disorders and evaluates both conventional and novel treatment approaches. By synthesizing current evidence, we aim to provide insights for developing more effective interventions to improve patient outcomes and quality of life.

## Simple Summary

Radiation therapy is a cornerstone treatment for brain tumors but frequently causes radiation-induced brain injury, damaging healthy brain tissue. A significant consequence of radiation-induced brain injury is the development of affective disorders, particularly major depression and anxiety, severely impacting patients’ quality of life. The underlying pathophysiology involves complex mechanisms like neuroinflammation, oxidative stress, blood–brain barrier disruption, and white matter damage. Current management relies on antidepressants, corticosteroids, and neuroprotective agents, while promising new therapies target neuroinflammation and neural repair pathways. This review examines the pathogenesis of radiation-induced brain injury-related affective disorders and evaluates both established and emerging treatment strategies, aiming to inform the development of more effective interventions to improve patient outcomes.

## 1. Introduction

Brain tumors are one of the most leading causes of cancer-related death globally, especially in children [1,2]. Metastatic brain tumors, typically from lung cancer, breast cancer and melanoma, jeopardize about 10 to 30 percent of adult cancer patients and 6 to 10 percent of childhood cancer patients [3,4]. At present, radiation therapy is the primary treatment for brain tumors. However, although the survival time of tumor patients has continuously extended with the rapid development of radiotherapy technology, the probability of radiation-induced brain injury (RBI) has also aroused the attention of doctors in the meantime [5,6,7,8,9]. Affective disorders such as depression and anxiety, generally appear several years after brain radiotherapy [10]. Specifically, depression is mainly manifested as low mood, insomnia, and fatigue [11]; anxiety disorders are primarily featured with stress and autonomic hyperactivity [12]. As the above symptoms all seriously affect the quality of patients’ life, RBI undoubtedly causes tremendous economic pressure not only on society but also on the medical and health environment.

The pathological mechanism of affective disorders after radiotherapy is complicated. Currently, the main hypotheses include chronic neuroinflammation, damage to emotion-related brain areas such as decreased hippocampal neurogenesis, lack of neurotrophic factors, synaptic ultrastructure impairment, and functional defects, etc.

The purpose of this review is to systematically examine the clinical manifestations, underlying mechanisms, and therapeutic approaches for RBI-associated affective disorders. This synthesis of current evidence will facilitate further investigation into RBI and its neuropsychiatric sequelae, with the ultimate goals of expanding treatment paradigms, optimizing clinical interventions, and improving patients’ quality of life.

## 2. The Association Between RBI and Symptoms of Depression and Anxiety

### 2.1. Clinical Trials

The current clinical trials are still in their early stages. As early as 1997s, a meta-analytical review conducted by Van’t Spijker et al. demonstrated that the incidence of depression in cancer patients was often higher than that in the general population [13]. Moreover, after Whole-brain radiation therapy, attention decline is common [14]; and this symptom is especially apparent in patients with attention problems [15]. The incidence of depression is 54%, and the mortality rate of cancer patients suffering from depression is twice that of other cancer patients [16]. A study that, respectively, examined the Baker Depression and Anxiety Survey of 13 brain tumor patients before and after radiation therapy showed that depression and anxiety were remarkably higher in those who were administrated with radiation therapy than those who were not [17]. A study conducted by Wang et al. in 2022 revealed a significant increase in the rates of depression and anxiety following radiation therapy for nasopharyngeal cancer. Specifically, the prevalence of anxiety among nasopharyngeal cancer patients increased from 34% prior to radiation therapy to 64% afterward, while the prevalence of depression rose from 25% to 56% [18]. Compared with adults, children receiving craniocerebral radiotherapy are more susceptible to severe depression and anxiety [18]. Another paired case-control study, which included 46 patients with NPS as the case group and 46 patients without NPS as the control group, found that the depression and anxiety levels in the case group were drastically higher than those in the control group [10]. Although clinical trials do not directly prove that RBI causes affective disorders, they suggest that RBI is a potential hazard for depression and anxiety in cancer patients and may be an underlying cause. Moreover, the prevalence of anxiety and depression was the highest in patients with head and neck tumors after radiotherapy [19]. Atrophy of certain brain area such as amygdala was proved to connect with poor memory, mood and emotional health. This pathological change may also happen during RBI. As expected, a recent longitudinal clinical trial showed bilateral amygdala and nucleus atrophy occurred in a time and dose-dependent manner after brain radiotherapy. Therefore, treatment plans that preserve the amygdala may preserve neurocognitive and neuropsychiatric functions in this population [20,21] (Table 1).

### 2.2. Animal Tests

During cancer diagnosis and treatment, patients will inevitably be bothered with negative emotions, which is likely to trigger psychiatric comorbidities [11]. For this reason, experimental models used to study depressive symptoms after cancer treatment have been carefully chosen. However, as early as 1994, Miyachi, Y., in the process of studying the effects of low-dose X-ray, found that mice showed significant behavioral inhibition after 5 to 15 cGy total body X-ray irradiation [22], but the higher dose (25–35 cGy) did not play the same role. This may be explained by the radiation dose-dependent genetic changes; brain tissue in mice after total-body irradiation transcriptome spectrum analysis showed that low-dose exposure (10 cGy) induced genes are not affected by high-dose radiation (2 Gy), and the gene induced by low dose also has its unique signal pathways and functions [23]. In 2014, Son et al. reported that mice exposed to a single 10 Gy irradiation showed significant depression-like behaviors at 30- and 90-day post-exposure [24]. However, in 2019, Ueno, H et al. irradiated the brains of mature mice with 20 Gy ionizing radiation to study the behavioral disorders of mice in the subacute stage. In the forced swimming experiment one week later, the irradiated mice had substantially less immobility time than the control group, and the mice showed depression-like behaviors compared with the control group [25]. All of these above animal experimentations provide evidence for RBI-related depression.

The results of animal experiments on depression caused by RBI are relatively simple, mostly showing an increase in depressive behavior. However, results from animal studies related to anxiety have been controversial, despite the significant stress generated by potential radiation toxicity in the brain [26]. In 2020, Dey, D. et. Al. reported that five weeks after radiotherapy combined with TMZ chemotherapy, the mice exhibited anxiety-like behaviors, and anxiety-like along with depression-like behaviors were observed at week 15. Notably, neither irradiation nor TMZ treatment alone induced significant anxiety-like behaviors [27]. In 2016, Brown, R. J. et al. administered fraction-dose or single high-dose whole-brain irradiation to young rats, and no significant difference was observed in open field tests during the subacute phase, but the change in exercise habits and anxiety-like behavior increased [28]. At the same time, Ueno, H. et al. irradiated the brains of mature mice with 20 Gy, and the tail suspension test showed no abnormality after 3–6 days, which indicated that severe anxiety behavior was unlikely to be caused in the subacute phase, but it may also be related to the dose of irradiation, as low-dose irradiation seems to be more likely to cause anxiety disorder compared with high dose [25]. In 2012, Trivedi, R. et al. reported that mice irradiated with radiation doses of 3, 5, and 8Gy evaluated the emotion-related behaviors. The results found that on day 5 post-irradiation, both the 3 Gy and 8 Gy doses exhibited antianxiety effects (as evidenced by prolonged center time), whereas the 5 Gy dose showed no significant effect. By day 10, anxiety-like behavior demonstrated a trend toward dose-dependent modulation; however, no statistically significant difference was observed between the 5 Gy and 8 Gy groups [29]. In 2017, Zhou K et al. found that the duration of staying in the open central area in the irradiated group (12 Gy) was 71.4% higher than that in the non-irradiated group after 28 days of birth [30]. The reasons accounting for above controversial results are complicated, including different experimental conditions (including radiation dose, type, time, etc.), variances in animal species, and lack of consistent anxiety-related behavior tests (Table 2).

## 3. Possible Mechanism of RBI Leading to Depression and Anxiety

Theoretically, brain necrosis will inevitably cause affective disorders when RBI affects cognition-related brain regions. Although the mechanism of RBI and its underlying molecular basis remain unclear, researchers have reached a consensus that RBI pathogenesis is complex, continuous, and dynamic. This process involves multiple aspects, including radiation-induced blood–brain barrier disruption, injury to various neural cell types, microvascular damage, neuroinflammation, impaired neurogenesis, and other contributing factors [35]. These complex mechanisms ultimately lead to diverse pathological manifestations, ranging from focal radiation necrosis to diffuse leukoencephalopathy with cerebral atrophy [36].

### 3.1. Hippocampal Dysfunction

The hippocampus is an important part of the cerebral limbic system, which plays a predominant role in cognitive and emotional regulation [37]. The dorsal part of the hippocampus mainly regulates learning and memory functions, while the ventral part is primarily responsible for emotional behavior and stress response [38,39]. It has also been confirmed that hippocampal dysfunction is associated with the development of neurological and psychiatric disorders [11]. Numerous data have demonstrated that craniocerebral radiation can induce hippocampal-dependent behavior disorders, including depression-like behavior [40,41].

#### 3.1.1. Reduced Hippocampal Neurogenesis

Adult neurogenesis persists throughout life, albeit with a gradual age-related decline. In the mature mammalian brain, neural stem cells are primarily located in the subventricular zone of the lateral ventricles and the subgranular zone of the hippocampal dentate gyrus [42]. Therefore, these two areas will differentiate into new neurons when stimulated. Radiotherapy can inhibit hippocampal neurogenesis, hence leading to numerous changes in the brain including a shift from long-term potentiation (LTP) to long-term depression and a permanent alternation in synaptic plasticity. And this pathological process is more pronounced in adolescents than in adults [43,44]. Some previous studies have suggested that changes in adult hippocampal neurons may be fundamentally involved in the pathophysiology of depressive symptoms [45,46,47]. Sustained neurogenesis is defined as the continuous development of new brain neurons, which is indispensable for maintaining the normal behavioral functions. When neurogenesis is inhibited through pharmacological means, individuals may experience symptoms commonly found in depressed patients [48,49]. This finding was further supported by a study, where Santarelli et al. discovered that antidepressants induced neurogenesis disruption prevented the expected behavioral responses to these medications [50]. NRBF2 is essential part of the autophagy-associated phosphatidylinositol 3-kinase complex, which contains PIK3C3/VPS34 [51]. Increasing the levels of NRBF2 in the adult neural stem cells of the DG region of the brain was able to reverse the negative effects on neurogenesis and alleviate symptoms of depression in the mice [52]. Overall, these findings highlight the importance of sustained neurogenesis for maintaining behavioral homeostasis and provide insights into the molecular mechanisms underlying the link between neurogenesis and depression. Although reduced neurogenesis is thought to be closely related to mood disorders, its exact mechanism of action remains controversial. Several animal studies have demonstrated a direct relationship between neurogenetic disorders in the hippocampus and depression under specific conditions of ionizing radiation. Earlier in 2011, Snyder et al., using either transgenic or radiation methods to inhibit adult neurogenesis specifically, found that mice with neurogenic defects showed increased immobility during forced swimming tests and decreased preference for sucrose compared to controls [53]. These findings confirm that neurons in the dentate gyrus are critical to the negative regulation of the HPA axis in the hippocampus and support the direct role of neurogenesis in post-radiation depression in adults. Son et al. (2014) reported that mice exhibiting depressive-like behaviors during the 90-day period following 10 Gy irradiation showed a progressive reduction in hippocampal doublecortin (DCX, an immunohistochemical marker of neurogenesis)-positive cells [24]. Kang, J. et al. also observed similar depressive behavior in the mouse model 30 days after 2 Gy skull irradiation, which was accompanied by a downregulation of the BrdU/Neun and DCX-positive populations, as well as an upregulation of MAP-2 expression in the hippocampus [32]. After analyzing the brain transcriptional files of radiation-induced depressed mice, they found that radiation-induced depressed mice increased expression of thyroxine transporter protein (TTR). This suggests that depression, one side effect of cranial radiotherapy, could be mediated by the inhibition of a pathophysiological procedure called retinol-regulated hippocampal neurogenesis [32]. However, in another study, hippocampal neurogenesis was reduced after a single dose of 5 Gy irradiation, but there was no significant change in the immobility time of the mice during the TST test [31]. The difference between them can be explained by differences in the type or dose of radiation. Therefore, further research is needed to determine whether hippocampal neurogenesis contributes to the improvement of depression-like behavior. At the same time, various factors, such as age, radiation source, dose, sex, and genes, as well as the duration of the behavioral studies, may influence the observation of behavioral changes. It is generally believed that women are more likely to be bothered with mood disorders. For instance, Roughton, K. et al. irradiated the whole brain of male and female mice with a dose of 8 Gy on the 14th day after birth. After ionizing radiation, the proliferation of neural stem cells in the subgranular region of the dentate gyrus of the hippocampus was sharply suppressed, especially in females, who were more inclined to behavior anxiously in the open field experiment. Therefore, there may be a long-term sex difference in craniocerebral irradiation [33]. In addition to gender, the role of genes has also become an increasingly hot research topic. In 2012, Brackett, J. Et al. reported that homozygous antioxidant enzyme related gene defects were more likely to produce delayed mood effect [54]. Another study demonstrated that female ApoE-deficient mice exhibited elevated anxiety-like behaviors [34]. These studies shed light on the potential genetic influence on radiation-induced affective disorders.

#### 3.1.2. Nerve Growth Factor

Brain-derived neurotrophic factor (BDNF), a member of the neurotrophic factor family, is abundant in the hippocampus and indispensable to the survival, proliferation, and differentiation of neurons [55,56]. During central nervous system development, neurotrophic factors are highly sensitive to radiation, so the regulation of BDNF and its receptor expression may be altered by very low doses of radiation (0.02 Gy) [57]. Moreover, the downregulation of BDNF mRNA or protein expression in hippocampus dentate gyrus of anxious or depressed patients is thought to be associated with psychiatric disorders [58,59,60]. This evidence indicates that BDNF play a potential role in hippocampal-dependent emotion regulation. Antidepressants exert therapeutic effects by inducing the expression of neurotrophic factors while targeting apoptotic proteins [61,62,63]. Accordingly, radiation may influence the emotion by altering BDNF levels as well [64]. As expected, a study showed that at 30 and 90 days post-10 Gy irradiation, the downregulation of hippocampal BDNF and GDNF mRNA levels coincided with the emergence of depressive behaviors in rats [24]. The levels of BDNF mRNA and protein in the hippocampus have been proved to be affected by various factors. However, there is still a lack of detailed study on how radiotherapy directly or indirectly affects BDNF mediated mood disorders. It is already well known that intestinal flora could mediate cognitive dysfunction according to the brain–gut axis. Interestingly, this mechanism may also apply to the radiation-induced affective disorders. For example, studies have shown that radiation therapy can affect the composition of intestinal flora, and probiotics can control the expression level of BDNF to alleviate anxiety behavior [38,65]. Therefore, one possible mechanism is that radiotherapy affects emotional regulation by altering the expression of BDNF in the hippocampus through the brain–gut axis.

#### 3.1.3. Alterations of Synaptic Plasticity

The interconnection of neural networks is indispensable to the normal central nervous system operation. The establishment and maintenance of such a network require precise regulation of neuronal dendrites and dendritic spine growth. The connections between them are dependent on the synaptic genesis. Apart from the morphology of the synapses that is composed of synapses structural plasticity and the number of synapses, the chemical plasticity is also closely related to synaptic density. Decrease in dendritic complexity, loss of synaptic connections, and impaired synaptic genesis are observed in major depressive disorder (MDD) patients [66,67,68]. The synaptic pathology charactered with synaptic plasticity reduction may play an important role in the onset and progression of affective disorders [67,69,70,71,72]. Many researches have confirmed that radiation can deteriorate synaptic plasticity [73,74]. For example, in 2022, Wu, M. Y. Et. Al. observed a remarkable reduction in peak discharge and excitatory synaptic inputs in hippocampal CA1 pyramidal neurons, while inhibitory inputs were greatly enhanced in the mice after receiving 10 min of cranial irradiation at a dose rate of 3 Gy/min. Corresponding to electrophysiological outcomes, they found suppressed expression of one synaptic plasticity marker called VGLUT1, a vesicular excitatory neurotransmitter transporter, along with the increased expression of VGAT, a vesicular inhibitory neurotransmitter transporter. In addition, in the irradiated mice, long-term enhancement of the hippocampus was diminished and GluR1 expression was suppressed, which suggested that radiation compromises not only the intrinsic excitability but also synaptic plasticity of hippocampal CA1 pyramidal neurons [75]. In 2013, Parihar, V. K. Et al. found that cranial radiation (1 and 10 Gy) drastically reduced the complexity of dendritic structures in mice 10 and 30 days after exposure. Crucial neuron morphology index including dendritic branches, length, and area degenerated in a dose-dependent way (>50%). Under the same dose and time, the structure of hippocampal dentate gyrus neuron dendritic spines was also seriously deteriorated, namely a 20–35% reduction in the number accompanied with a 40–70% decrease in density [76]. In contrast to the previously found long-term loss of dendritic spines, Duman, JG et al. in 2018 demonstrated that the acute response to radiotherapy was an increase in spinous processes and excitatory synapses before a spinal synaptic density reduction. Therefore, reversing the synaptic remodeling may become a promising therapeutic strategy for preventing the acute radiation-induced toxicity, and the memantine is one of the representative drugs [77]. Several previous researches have demonstrated that cranial IR alters LTP, N-methyl-D-aspartate receptor subunits, and glutamine transportability, which all play essential roles in maintaining synaptic plasticity [43,78,79]. Similarly, the abnormal glutamatergic system featured with different postsynaptic NMDA (N-methyl-D-aspartic acid) and AMPA (α-amino-3-hydroxy-5-methyl-4-isoxalo-propionic acid) receptor subunits expression in the hippocampus and prefrontal cortex (PFC) has also been illustrated in a rat model of depression [80]. Pro-inflammatory cytokines released after radiation, such as TNF-α and IL-1, can have a direct function on neurons via binding to the extracellular domain of the cytokine receptor and then activating downstream signaling pathways, thereby altering excitability, synaptic strength, and synaptic scaling [81,82,83]. RBI may affect the transduction of neurotrophic factors and related signals by interfering with synaptic plasticity, thus contributing to post-IR mood disorders.

### 3.2. Chronic Inflammation

Although the specific mechanism of RBI remains unclear, it is generally believed that chronic inflammation and oxidative stress play an essential role in the occurrence and progression of RBI. During the inflammation, no matter acute or chronic phase, the mRNA expression of pro-inflammatory mediators, such as tumor necrosis factor-α (TNF-α), Interleukin (IL-1β, IL-4, IL-6, and IL-8), Monocyte chemoattractant protein 1 (MCP-1), Inducible nitric oxide synthase (iNOS) and Intercellular adhesion molecule 1 (ICAM-1) was substantially upregulated in the mice brain [83,84,85]. Increased levels of interleukin-6 (IL-6), interleukin-1β (IL-1β), CCl2, C-reactive protein (CRP), and TNF-α in the blood and cerebrospinal fluid were also detected in the patients haunting by depression. The “depression cytokine hypothesis” implies that these inflammatory cytokines play a crucial part in developing depression [86,87,88,89,90,91]. According to the above interesting hypothesis, alternations in cytokine-induced reward pathways and dopamine neurotransmission may be the underlying mechanism of mood disorders after RBI.

Brain irradiation triggers an inflammatory storm including the over-activation of microglia and excessive secretion of inflammatory cytokines by macrophages [88,92]. The total population of microglia decreased after IR, while the activated microglia upregulated. Similar results were also found in the postnatal rats 7 days after a single 8 Gy brain irradiation and in the elderly rats receiving 10 Gy whole-brain radiation [93,94]. And there is evidence that depression could be deemed as a microglia-mediated chronic neuroinflammation disease [95,96,97]. Setiawan, E. et al. found that in MDD patients, transporter density, as measured by distributed volume (TSPO VT), increased in activated microglia, which is a distinctive feature of neuroinflammation [96]. Therefore, one of the possible mechanisms is the activation of microglia induced by radiation-induced chronic inflammation. The decreased levels of neurotrophic factors in inactive microglia combined with the activated microglia stimulated by pro-inflammatory mediators lead to hippocampal dysfunction. Activation of microglia also modulates noradrenergic and serotonergic neurotransmission and can induce depressive- and anxiety-like behaviors through the NLRP3 inflammasome/IL-1β signaling pathway [97,98,99]. Additionally, behavioral tests supplemented by magnetic resonance imaging and immunohistochemistry yielded consistent results showing radiation-induced post-necrotic mood disorders and microglial activation in the right primary somatosensory cortex of Fischer rats [100]. The decline in the number of microglia in adult mouse brains 30 and 90 days after a single 10 Gy irradiation was consistent with the occurrence of depression-like behavior [24]. In a word, activated microglia may be one of the crucial causes of mood disorder after radiotherapy.

Ionizing radiation produces reactive oxygen species (ROS) that break DNA double strands, followed by the spread of inflammatory responses involving cytokines and chemokines [36]. Inflammation and oxidative stress are concerned with the injury of glutamate neurotransmitters. Astrocytes release chemical transmitters (including glutamate) in a calcium-dependent manner. Cytokines can suppress the expression of glutamate transporters in astrocytes and increase the release of glutamate [101]. Notably, the glutamate released by astrocytes preferentially binds to the extracellular domain of NMDA receptors, leading to reduced production of trophic factors (including BDNF), ultimately compromising neuronal integrity [102,103]. Associated with depression is increased glutamate content in the frontal cortex of patients with mood disorders, along with evidence of increased microglia activation and QUIN expression [98,104,105,106]. Dysregulation of inflammatory cytokine production and signaling in the brain can contribute to cognitive and emotional deficits by activating inflammation-related enzymes such as indoleamine 2,3-dioxygenase (IDO), which are expressed by activated astrocytes, microglia, and infiltrating macrophages [107,108,109]. IDO is a tryptophan degradation enzyme, and inflammatory cytokines can enhance its activity. In chronic inflammation, the over-activated IDO accelerate the consumption of tryptophan, one raw material for serotonin synthesis. Activation of IDO facilitates the tryptophan metabolic reprogramming that converts the tryptophan into the kynurenine pathway instead of generating serotonin, producing neurotoxic metabolites such as 3-hydroxykynurenine, quinolinic acid [108,110,111,112]. Besides their stimulant toxicity, these anxiety- and depression-related metabolites also activate monoamine oxidase (MAO), the enzymatic target of antidepressants that degrades serotonin (5-HT), dopamine (DA), and norepinephrine (NE) [90,113]. In the pathogenesis and treatment of depression, IDO have the potential to become a new pharmacological research target. However, whether it is a crucial factor or just a participating factor in the complex mechanism of depression remains to be further studied. This association has not been observed in radiation-induced animal models. However, clinical studies have shown significantly increased IDO activity (calculated by the Kyn/Trp ratio) in non-small cell lung cancer patients after receiving a biologically equivalent dose (BED) > 70 Gy, compared to pre-radiotherapy levels [114]. Similarly, cytokines may also disturb the synthesis of dopamine DA by reducing the bioavailability of tetrahydrobiopterin (BH4). BH4, a key enzyme cofactor involved in the production of dopamine DA, is highly sensitive to REDOX and easily oxidized to dihydrobiopterin (BH2). What is more, this process is irreversible [115,116]. Cytokines also have an impact on dopamine packaging, release, and reuptake. Dopamine relies on vesicular monoamine transporter 2 (VMAT2) to encapsulate cytoplasmic dopamine for release. There is evidence that inflammatory cytokines (IL-1β and TNF-α) can impair VMAT2 function [117]. It is worth noting that mitochondrion, an important oxidative stress victim, has also become a significant target for inflammation-related damage. The upregulation of radiation-induced pro-inflammatory factors (including IL-6, TNF-α, and ROS) impairs mitochondrial function and induces interferon-α (IFN-α) production, which exhibits both neuropsychological and neurotoxic effects [118,119,120,121]. These factors together influence hippocampal neurogenesis and lead to behavioral dysfunction. Studies have demonstrated that decreasing levels of TNF-α, IL-6, iNOS, and IL-1β can mitigate neuropsychiatric symptom severity. Furthermore, inhibition of the STAT3/NF-κB pathway activity—a key inflammatory regulatory pathway—also ameliorates these symptoms [122]. More detailed researches are needed to understand the mechanisms of radiation-induced neuroinflammation and its exact role in affective disorders.

### 3.3. Other Possible Mechanisms

In addition, there are some other mechanisms, including vascular damage, nerve demyelination, and endocrine changes, caused by RBI [123,124]. Brain irradiation exerts profound effects on the cerebrovasculature, potentially leading to delayed radiation-induced necrosis [125]. Ten to fifty-two weeks after irradiation, the dose of 5 Gy/ fraction (8 fractions in total) can reduce the endothelial cells population, vascular density, and vascular length [126]. Blood–brain barrier permeability and vascular endothelial growth factor (VEGF) expression increased within 24 h after exposure to 6 Gy radiation [127]. Inflammatory factors induced by radiotherapy, such as iNOS, also increase BBB permeability [128]. The high permeability of the blood–brain barrier enables the peripheral inflammatory cytokines enter the cerebrospinal fluid and these inflammatory factors in turn impair the integrity of the blood–brain barrier. This positive feedback loop results in a cascade neuroinflammatory response, finally leading to emotional disorders.

In a model of chronic depression induced by stress, loss of myelin sheath may play an indispensable role shown by diffusion tensor imaging [129]. Also, several studies have demonstrated that depression and anxiety are common symptoms of demyelinating diseases such as epilepsy and multiple sclerosis [130,131,132]. One of the main pathological manifestations of RBI is the demyelination of oligodendrocytes, so it is speculated that radiotherapy can cause affective disorders through demyelination as well [36]. Conpanzo et al. found that ruptured and demyelinated splenial fiber bundles in the right hemisphere of Fischer rats 54 days post-irradiation, along with selective behavioral changes (including depressive- and anxiety-like behaviors), could be attributed to these ultrastructural lesions [100]. In summary, the underlying mechanism of emotional change can be ascribed to the neuron connectivity alternations between the limbic area and the PFC [129].

Radiotherapy can distinctly reduce the secretion of melatonin in the pineal gland of patients, aggravating severe sleep difficulties and prevalence of depression [133]. Therefore, the occurrence of depression and anxiety may be explained by the post-radiotherapy neuroendocrine changes. Studies have reported that radiation affects the functions of the growth hormone/insulin-like growth factor-I axis, restricting physical and mental development [134]. Moreover, radiation can markedly reduce the rate of glucose metabolism in the temporal and occipital lobes. Therefore, brain metabolic suppression is proposed to become one of the possible ways to induce metabolic depression [135]. For example, depression and anxiety are representative clinical manifestations of post-cranial irradiation somnolence syndrome [136,137]. The pathophysiology of somnolence syndrome may be associated with the transient demyelination of white matter or the nonspecific effects of chronic diseases and poor emotional perception.

## 4. Potential Diagnostic, Assessment, and Preventive Strategies

### 4.1. Early Neuropsychological Assessment

Traditional psychiatric assessment tools, such as the hospital anxiety and depression scale (HADS), are effective in screening for anxiety and depressive symptoms caused by radiation-induced brain injury. However, they exhibit insufficient sensitivity in detecting subclinical cognitive dysfunctions resulting from such damage. Given that the onset of radiation-induced anxiety and depression is closely associated with hippocampal dysfunction, evaluating hippocampal injury plays a crucial role in the early diagnosis of these conditions.

The Wechsler memory scale-revised (WMS-R) is a widely used neuropsychological tool for assessing an individual’s memory function, including short-term memory, long-term memory, and working memory [138]. It measures various memory dimensions through multiple core subscales, such as attention, general memory, visual memory, verbal memory, and delayed memory [139]. Its standardized scoring system uses the memory quotient (MQ) to quantify results, with an MQ score of 80 or above indicating normal functioning. Scores between 70 and 79 suggest a borderline condition, while scores of 69 or below indicate impairment. Moreover, lower scores correspond to more severe deficits. The WMS-R has significant clinical value in evaluating hippocampal damage [140]. Numerous studies have found that patients with hippocampal damage often have very low WMS-R scores, particularly in delayed memory (less than 50) [141,142,143]. The California verbal learning test (CVLT) is a widely used tool in clinical and neuropsychological assessments, primarily aimed at evaluating an individual’s verbal learning and memory abilities [144]. This test involves a series of tasks, including immediate recall, interference list, short-delay free recall, and cued recall, to assess memory performance and learning strategies at various stages [144]. The CVLT holds substantial practical value in clinical settings [145]. It aids clinicians in identifying and evaluating various neuropsychological disorders, such as Alzheimer’s disease (AD), traumatic brain injury, and others [146,147].

Although no studies have yet specifically applied the WMS-R and the CVLT for the early identification of RBI and its associated anxiety and depression, these neuropsychological assessment tools hold significant potential for future applications. Given their role in assessing hippocampal dysfunction, they may be valuable in the early detection of RBI and related psychological symptoms.

### 4.2. Advanced Imaging Techniques

With the rapid advancement of neuroimaging technologies, many advanced imaging techniques have provided new breakthroughs in understanding the mechanisms of radiation-induced brain injury. These techniques offer unique insights into the structural and functional abnormalities of the hippocampus, providing important evidence for clinical diagnosis, evaluation, and treatment planning.

Resting-state functional magnetic resonance imaging (rs-fMRI) detects spontaneous neural activity in the brain, enabling precise assessment of the functional connectivity between the hippocampus and the default mode network [148]. Task-based fMRI, on the other hand, directly observes hippocampal functional activation patterns by designing specific memory encoding or emotional processing tasks [149]. In patients with AD, rs-fMRI can detect early abnormalities in hippocampal activity, while task-based fMRI demonstrates characteristic hippocampal activation reduction, often preceding clinical symptoms [150,151]. Additionally, studies have shown that after whole-brain radiotherapy (WBRT), patients exhibit widespread hyperconnectivity in rs-fMRI, along with significant increases in global modularity and local network variability, which are strongly correlated with cognitive decline [152].

Arterial Spin Labeling (ASL) Perfusion Imaging provides a new dimension for assessing the hippocampus. This technique does not require exogenous contrast agents and allows for the repeated measurement of cerebral blood flow (CBF) in the hippocampal region [153]. In patients with radiation-induced brain injury, ASL-CBF values are typically lower, indicating reduced cerebral blood perfusion [154]. Magnetic Resonance Spectroscopy (MRS) quantifies the concentration of metabolites such as N-acetylaspartate (NAA) and myo-inositol, which can sensitively reflect the degree of neuronal damage [155]. MRS exhibits higher detection sensitivity for radiation-induced brain injury, with a characteristic increase in the NAA/Choline (Cho) ratio in affected patients [156,157]. Fluorodeoxyglucose Positron Emission Tomography (FDG-PET) detects changes in glucose metabolism and can identify early hippocampal functional abnormalities [158]. Studies have shown that a reduction in central nervous system glucose metabolism following brain radiotherapy is closely associated with cognitive decline [159].

Advanced imaging technologies hold significant value in the clinical assessment of radiation-induced brain injury. By integrating these advanced techniques, a comprehensive evaluation of the neurobiological changes in brain injury can be achieved from multiple dimensions, thereby significantly enhancing the sensitivity and specificity of early diagnosis [152,154,156,157,159]. The complementary application of these techniques not only helps to distinguish radiation-induced brain injury from neurodegenerative diseases such as AD but also provides objective evidence for treatment monitoring and prognostic assessment [150,151,152]. In the clinical context of radiation-induced brain injury-related anxiety and depression, these advanced imaging techniques can be applied to patients with depressive and anxiety symptoms following brain radiotherapy. They reveal the specific neurobiological alterations in such patients, enabling effective differentiation between secondary and primary mood disorders and predicting the risk of symptom progression, thus offering objective support for personalized treatment strategies [160].

### 4.3. Hippocampus Avoidance Radiotherapy Strategy

Hippocampal Avoidance Whole-Brain Radiotherapy (HA-WBRT) is an innovative radiation therapy technique designed to reduce the risk of cognitive dysfunction induced by conventional WBRT by precisely avoiding the hippocampal region. [161]. This technique primarily relies on advanced radiation therapy methods, such as Intensity-Modulated Radiotherapy (IMRT). IMRT dynamically adjusts the intensity distribution of the radiation beams to ensure adequate dose delivery to the tumor target while minimizing exposure to critical neural structures, such as the hippocampus [162,163]. In clinical practice, the hippocampal anatomy is accurately located using MRI-CT fusion imaging, and a 5–10 mm safety margin is set around it as the avoidance region. Treatment planning was performed to deliver 30 Gy in 10 fractions to the whole brain while maintaining hippocampal dose below protocol-specified limits (D100% < 9 Gy, Dmax < 16 Gy) in accordance with RTOG 0933 guidelines [164]. Studies have shown that this approach can reduce the average radiation dose to the hippocampus by up to 87%, significantly outperforming traditional radiotherapy techniques [163]. Furthermore, other technologies such as Volumetric Modulated Arc Therapy (VMAT) can also be utilized within HA-WBRT to enhance treatment precision and efficiency [165].

In terms of neurocognitive protection, HA-WBRT has demonstrated clear short-term and long-term cognitive benefits [161]. Studies have shown that no significant cognitive improvement is observed within two months post-radiotherapy, but protective effects gradually emerge starting from three months [161,166]. At three months, significant differences were noted in memory/verbal learning (*p* < 0.0001) and overall cognitive function (*p* = 0.001) [166,167,168]. At six months, improvements in memory/verbal learning continued (*p* < 0.1) [161,167,169], and at 9–24 months, overall cognitive function maintained a significant advantage (*p* < 0.05) [168,170]. It is noteworthy that the improvement in memory function shows subtype specificity, with long-term memory benefiting more significantly than short-term memory [171]. Additionally, different cognitive dimensions exhibit varied responses to HA-WBRT. This technique is particularly effective in enhancing overall cognitive function, memory, and verbal learning, but improvements in executive function, visual learning, attention, and information processing speed are relatively limited [172].

In terms of survival outcomes, HA-WBRT demonstrates comparable efficacy to conventional WBRT. Long-term follow-up data from several key clinical trials confirm that there are no significant statistical differences between the two treatment modalities in key survival indicators, such as overall survival (OS) and progression-free survival (PFS) [161,167,169,173]. These findings strongly suggest that, despite the significant reduction in radiation dose to the hippocampal region, HA-WBRT does not compromise tumor control. The clinical evidence provides strong support for the dual goals of HA-WBRT, achieving both neurocognitive protection and survival benefits.

Although existing studies primarily focus on cognitive outcomes, given the hippocampus’s close involvement in emotional regulation, HA-WBRT may theoretically improve mood symptoms by preserving hippocampal structural integrity [174]. However, direct evidence for improvements in depression and anxiety is currently lacking, and prospective studies combining emotional scales with hippocampal volume/function MRI parameters are needed for systematic evaluation. Overall, HA-WBRT represents a significant advancement in neuroprotective radiotherapy, and its potential value in emotional regulation suggests that it could serve as a promising strategy for preventing radiation-induced brain damage leading to depression and anxiety. Further validation of its comprehensive clinical benefits through multi-center randomized controlled trials is warranted.

## 5. Potential Treatments

While multiple therapeutic interventions exist for RBI, current evidence regarding their efficacy in managing associated depression and anxiety remains limited. This review systematically evaluates pharmacological agents with demonstrated effectiveness in treating RBI-related mood disorders.

### 5.1. Inhibitors of Glycogen Synthase

Glycogen synthase kinase 3 (GSK-3) is a serine/threonine kinase composed of two subunits, namely GSK-3α and GSK-3β [175,176]. It mainly phosphorylates Tau protein to increase Tau protein content in neuronal fibers and causes neuronal defects together with β-amyloid plaques, which are associated with neurodegenerative diseases such as Alzheimer and Parkinson [177,178]. As a result, GSK-3 has emerged as a promising therapeutic target for neurodegenerative diseases. Recent studies have demonstrated the efficacy of synthetic GSK-3 inhibitors in mitigating AD, Parkinson’s disease (PD), mood disorders, and other neurological conditions [179,180,181]. Lithium is the first identified GSK3 inhibitor, which can inhibit stem cell or progenitor cell death, attenuate neuroinflammation, improve synaptic plasticity, stimulate stem cell and progenitor cell proliferation and neurogenesis, and thus play a powerful neuroprotective role after cranial irradiation [182,183,184,185,186,187]. Even better, lithium not only does not protect cancer cells against radiotherapy but may serve as a radiation sensitizer in some cases [188,189]. In a radiation-induced rodent model, lithium treatment attenuated radiation-induced progenitor cell death in the hippocampal subgranular zone, enhanced neurogenesis and astrocytogenesis in juvenile rats, and ameliorated radiation-associated anxiety-like behaviors [30]. Thus, glycogen synthase inhibitors are undoubtedly effective agents for treating post-radiotherapy mood disorders, but further investigations are still needed to elucidate the efficacy of drugs other than lithium.

### 5.2. Fluoxetine

Fluoxetine, a selective serotonin reuptake inhibitor (SSRI) antidepressant, has been widely used to treat the adult major depressive disorder, obsessive-compulsive disorder, bulimia neuropathy in clinical. Also, fluoxetine may play a protective role in cognitive disorders such as vascular dementia through increasing neurogenesis in the dentate gyrus of the hippocampus, enhancing synaptic plasticity, and upregulating the expression of BDNFs in the hippocampus [190,191,192,193]. A study reported that 5HT1A serotonin receptor levels declined by 50 percent in both early and late hippocampi, and a reduction of 37 percent in serotonin levels was observed at 15 weeks after radiotherapy. Chronic fluoxetine treatment was adequate to reverse depression-like behavior induced by the combined chemoradiotherapy [27]. One study found that mice with 6 weeks temozolomide treatment after single cranial irradiation of 9Gy spent less time in open arms during the elevated plus maze tests, whereas fluoxetine reversed this anxiety-like behavior [194]. Although studies have confirmed the efficacy of fluoxetine in alleviating post-radiation anxiety, the potential impact of temozolomide in combined interventions should not be overlooked. Further research is needed to clarify the relationship between post-radiation anxiety and fluoxetine response and to elucidate its underlying mechanisms. Additionally, certain side effects warrant careful consideration, including sexual dysfunction, manic switching, and even persistent pulmonary hypertension [195,196].

### 5.3. Allantoin and Neferine

Retinol and its major metabolite retinoic acid (RA) play a crucial role in hippocampal neurogenesis, anxiety-like behavior, and hippocampal-dependent memory in adulthood [197,198,199,200]. Moreover, dysregulated retinol signaling pathway is associated with neurological diseases including cerebral ischemia and AD, which are featured with age-related memory decline, long-term hippocampal enhancement, and neurogenesis [201,202,203,204]. Overexpression of TTR inhibits retinol-mediated neurogenesis. Allantoin and neferine, the active constituents of lotus, enhance retinol-mediated neuronal maturation via repressing TTR expression and promoting PAK1 phosphorylation. Studies have shown that these toxic-free natural substances can relieve depressive symptoms after craniocerebral radiotherapy. With the treatment of allantoin and neferine, the amount of BrdU^+^/Neun^+^ in irradiated mice dramatically reduced, which enhanced hippocampal neurogenesis and relieved neurodegeneration, thus alleviating the depressive symptoms caused by radiation [32]. Allantoin and neferine may serve as potential therapeutic agents for depression, potentially exerting antidepressant effects by mitigating hippocampal neurodegeneration caused by cranial irradiation.

### 5.4. Other Potential Treatment

Minocycline is a clinically available antibiotic that is charactered with neuroprotective ability in various neurodegenerative diseases through its direct antioxidant activity [205,206]. Since ionizing radiation triggers caspase-3-dependent apoptosis primarily through ROS generation, ROS scavengers such as minocycline can inhibit this apoptotic pathway in neural stem cells. Studies have shown that minocycline can significantly alleviate radiation-induced cognitive impairment and defend newborn neurons against radiation-induced apoptosis, thereby reducing the loss of new neurons [207]. In recent years, it has also been reported that minocycline can also treat emotional disorders. And inhibitory caspase-1 can regulate intestinal microorganisms to affect the inflammatory signal regulation pathway and then indirectly block the activation of IDO, thereby changing brain function and affecting depression and anxiety-like behaviors [208,209]. Therefore, it can be speculated that minocycline may be an effective drug for treating the post-radiotherapy affective disorder. P7C3 compounds are one type of neuroprotective agents that enhance hippocampal neurogenesis and have a more predominant neurogenic effect than antidepressants on the market. Its neuroprotective effects have been shown to protect people from cognitive deficits and depression-like behavior by promoting neurons to survive independent of early disease-specific pathology [210]. The study found that P7C3 compounds exhibited antidepressant effects in mice exposed to chronic social frustration, stress or caloric restriction, but ablation of hippocampal nerves by cranial irradiation could eliminate this antidepressant effect [211]. However, further clinical and animal experiments are needed to explore and prove whether it can exert an anti-depressive effect after radiotherapy. In addition to conventional medical treatments, mindfulness-based interventions (MBIs) have demonstrated significant efficacy in managing anxiety and depression symptoms among adult cancer patients. Notably, the 2023 ASCO Clinical Practice Guideline for managing anxiety and depression in adults with cancer strongly recommends various complementary interventions—including MBIs, yoga, relaxation techniques, music therapy, reflexology, and aromatherapy (via inhalation)—for alleviating anxiety symptoms during active cancer treatment. Furthermore, these non-pharmacological approaches are widely recognized for their sustained benefits in anxiety management during post-treatment survivorship care [19]. In addition to the above-mentioned interventions, the ASCO Guideline also suggests the use of MBIs, yoga, acupuncture, tai chi, and/or Qigong, along with reflexology, for treating anxiety symptoms after cancer treatment. Furthermore, for depressive symptoms during cancer treatment, the ASCO Guideline recommends the integration of MBIs, yoga, music therapy, relaxation techniques, and reflexology. These interventions have been shown to be effective in relieving symptoms of depression and promoting emotional well-being during the challenging period of cancer treatment. After cancer treatment, the ASCO Guideline emphasizes the importance of continuing MBIs, yoga, tai chi, and/or Qigong as potential treatments for depressive symptoms. In a word, these mind–body practices not only provide emotional support but also contribute to overall recovery and improved quality of life for cancer survivors in a subtle way [212].

## 6. Conclusions

Brain radiotherapy induced affective disorders such as depression and anxiety are predominant symptoms of the RBI. While many possible mechanisms including hippocampal dysfunction and chronic neuro-inflammation have been proposed, the research on its exact mechanism is still insufficient, and we need to rule out the spirit of comorbidities. Other confounding factors require further study to determine whether there is a causal relationship between them. Although it is hard to figure out which mechanisms directly contribute to affective disorders following radiotherapy, this review may provide insights and identify the underlying molecular mechanisms. Future research directions may include developing new protective agents to prevent or mitigate adverse reactions in patients with RBI. This review helps provide new targets for preventing emotional disorders caused by RBI to improve patients’ quality of life and extend life expectancy.

## Figures and Tables

**Table 1:** Clinical trials.

Study Design	Population Characteristics	Assessment Timepoints	Psychological Scales	Key Findings	Follow-Up Duration	Reference
Case-control	13 WBRT patients vs. 13 healthy controls	1-month post WBRT	BDI, BAI	The WBRT group showed significantly higher scores on the BDI (16.40 ± 12.16 vs. 4.00 ± 2.38) and BAI (14.47 ± 11.96 vs. 4.54 ± 3.30) compared to the control group (*p* < 0.05)	Weekly during treatment	[17]
Retrospective cohort	232 newly diagnosed NPC patients	Pre-RT, week 4, RT completion	HADS	Anxiety incidence: 34.0% → 55.1% → 64.0% (*p* < 0.001); Depression incidence: 25.0% → 43.9% → 56.0% (*p* < 0.001)	N/A	[18]
Case-control	46 RBI vs. 46 non-RBI NPC patients	Case group: post-RT: 6.0 ± 3.5 Control group: post-RT: 5.7 ± 3.1	SAS, SDS	The RBI group had higher incidence rates of depression (84.8%) and anxiety (87.0%). The SDS (63.48 ± 8.11 vs. 58.67 ± 7.52, *p* = 0.008) and SAS (67.36 ± 10.41 vs. 60.34 ± 9.76, *p* = 0.005) scores were significantly higher in the RBI group	N/A	[10]
Cross-sectional	100 cancer patients undergoing RT	Pre-RT, mid-RT, RT completion	HADS	Maximum prevalence of anxiety and depression was seen in patients having head and neck malignancies	N/A	[19]

RBI: Radiation-induced brain injury; SDS: Self-rating Depression Scale; SAS: Self-rating Anxiety Scale; BDI: depression inventory; BAI: Beck anxiety inventory; HADS: Hospital Anxiety and Depression Scale; WBRT: Whole-brain radiotherapy; RT: Radiotherapy.

**Table 2:** Animal tests.

Species	Age	Time After WBI	Radiation Dose	Radiation Type	Type of Experiment	Results	Reference
Male Fischer rats	28 days	4 months	27 Gy in 9 daily fractions of 3 Gy, or 34 Gy, where the last 3 Gy fraction being replaced with a 10 Gy boost	X-ray	EPM	The experimental group spending less time in the open arms relative to controls (27Gy: *p* = 0.002, 34Gy: *p* = 0.02).	[28]
Male strain A mice	6–10 weeks	0, 5, 10 days	3 Gy/single 5 Gy/single 8 Gy/single	γ-ray	OFT	On day 5 post-irradiation (PI), an anxiolytic effect was observed at the 3 Gy and 8 Gy dose levels, as evidenced by a significant increase in time spent in the center compared to controls. In contrast, the 5 Gy group showed no statistical difference from the control group. By day 10 PI, anxiety-like behavior exhibited an approximately dose-dependent response. However, no significant difference was detected between the 5 Gy and 8 Gy dose groups.	[29]
Male Wistar rat pups	11 days	28, 60 days	6 Gy/single	X-ray	OFT	The time spent in the open central zone was 71.4% greater in the irradiated rats compared to the non-irradiated controls (*p* < 0.05).	[30]
Male C57BL/6 mice	11 weeks	EPM, OFT: 3 days FST, TST: 6 days	20 Gy/single	X-ray	EPM OFT TST FST	EPM: There were no significant differences between irradiated and control mice in the total distance traveled (*p* = 0.8345), number of total entries into the arms (*p* = 0.4982), or the time spent in the open arms (*p* = 0.7938). OFT: Irradiated mice had significantly lower total distance and time spent in the central area compared with control mice (*p* = 0.0128; *p* = 0.0056). There was a significantly lower number of entries to the central area by irradiated mice than by control mice (*p* = 0.0346). FST: Irradiated mice spent significantly less time immobile in each 1 min period during the 10 min test period than did control mice on day 1 (*p* = 0.0106). However, on day 2, there were no significant differences in the percentage of immobility between irradiated and control mice (*p* = 0.9824). TST: There were no significant differences between irradiated and control mice (*p* = 0.3913; *p* = 0.4321).	[25]
Male C57BL/6 mice	8 weeks	1, 3 months	10 Gy/single	γ-ray	TST	At 30 days (*p* < 0.01) and 90 days (*p* < 0.001) post-irradiation, mice treated with 10 Gy showed significantly longer immobility times than sham-irradiated controls.	[24]
Female C57BL/6J mice	4 weeks	2 weeks, 2.5 months	5 Gy/single	X-ray	TST	WBI did not significantly alter total immobility time.	[31]
Male C57BL/6 mice	6 weeks	30 days	2 Gy/single	X-ray	TST FST	TST: Mice exposed to radiation displayed a significant increase in immobility duration in the TST at 30 days post-irradiation compared to non-irradiated controls (*p* < 0.0001). Additionally, irradiation markedly reduced active twisting and curling movements while prolonging passive swaying time during mobile phases. FST: Irradiated mice exhibited significantly prolonged immobility at 30 days post-exposure (*p* < 0.0001) relative to sham-treated controls.	[32]
Male and female C57BL/6J mice	14 days	4 months	8 Gy/single	X-ray	OFT	A significant reduction in center zone time was observed in irradiated female mice (*p* = 0.025), but not in males (ns).	[33]
Female C57BL/6J mice	6 months	3 months	10 Gy/single	γ-ray	OFT	There were no significant differences between irradiated and control mice.	[34]

OFT: Open Field Test; TST: Tail Suspension Test; FST: Forced Swim Test; EPM: Elevated Plus Maze Test; ns: not significant (*p* > 0.05).

## Data Availability

All the data cited in this review are from published literature. The relevant original data can be obtained through corresponding papers or public databases. No new experimental data were generated in this study.

## Abbreviations

The following abbreviations are used in this manuscript:

RBI	Radiation-induced brain injury
LTP	Long-term potentiation
TTR	Thyroxine transporter protein
BDNF	Brain-derived neurotrophic factor
MDD	Major depressive disorder
PFC	Prefrontal cortex
TNF-α	Tumor necrosis factor-α
MCP-1	Monocyte chemoattractant protein 1
iNOS	Inducible nitric oxide synthase
ICAM-1	Intercellular adhesion molecule 1
IL-6	Interleukin-6
IL-1β	Interleukin-1β
CRP	C-reactive protein
ROS	Reactive oxygen species
IDO	Indoleamine 2,3-dioxygenase
MAO	Monoamine oxidase
DA	Dopamine
NE	Norepinephrine
BED	Biologically equivalent dose
VMAT2	Vesicular monoamine transporter 2
IFN-α	Interferon-α
VEGF	Vascular endothelial growth factor
HADS	Hospital anxiety and depression scale
WMS-R	Wechsler memory scale-revised
MQ	Memory quotient
CVLT	California verbal learning test
AD	Alzheimer’s disease
rs-fMRI	Resting-state functional magnetic resonance imaging
WBRT	Whole-brain radiotherapy
ASL	Arterial Spin Labeling
CBF	Cerebral blood flow
MRS	Magnetic Resonance Spectroscopy
NAA	N-acetylaspartate
Cho	Choline
FDG-PET	Fluorodeoxyglucose Positron Emission Tomography
HA-WBRT	Hippocampal Avoidance Whole-Brain Radiotherapy
IMRT	Intensity-Modulated Radiotherapy
VMAT	Volumetric Modulated Arc Therapy
OS	Overall survival
PFS	Progression-free survival
GSK-3	Glycogen synthase kinase 3
PD	Parkinson’s disease
SSRI	Selective serotonin reuptake inhibitor
RA	Retinoic acid
MBIs	Mindfulness-based interventions
